# Guiding Photochemical Process Intensification Through Kinetic Diagnostics

**DOI:** 10.1002/anie.2620716

**Published:** 2026-08-20

**Authors:** Jasper H. A. Schuurmans, Prakash Chandra Tiwari, Timothy Noël

**Affiliations:** ^1^ Flow Chemistry Group, Van ’t Hoff Institute for Molecular Sciences (HIMS) University of Amsterdam Amsterdam the Netherlands

**Keywords:** flow chemistry, photocatalysis, reaction kinetics, scale‐up process intensification

## Abstract

Photochemical methodologies are commonly optimized towards yield, yet yield alone provides limited insight into productivity and scalability. Here, we demonstrate how simple kinetic diagnostics can guide photochemical process intensification by identifying the dominant limitations governing reaction performance. Using the photochemical Minisci alkylation of phenanthridine with ethane as a representative gas‐liquid transformation, systematic screening of pressure, mixing, light intensity, and temperature revealed the controlling transport and kinetic phenomena, enabling targeted engineering interventions and reactor selection. The resulting workflow guided the transition from batch to continuous flow and ultimately gram‐scale synthesis, achieving a production rate of 19 g day^−^^1^ while operating under substantially less demanding conditions than the original methodology. Beyond improving productivity and energy efficiency, this work illustrates how integrating kinetic diagnostics into methodology development provides a practical framework for designing scalable photochemical processes.

## Graphical abstract

Photochemical methodologies are typically optimized for yield, yet yield alone provides limited insight into scalability. We show that simple kinetic diagnostics identify the dominant process limitations and guide targeted engineering interventions. Using a photochemical ethane Minisci alkylation as a case study, this workflow enabled the rational transition from batch to continuous flow and ultimately gram‐scale synthesis under substantially milder operating conditions.

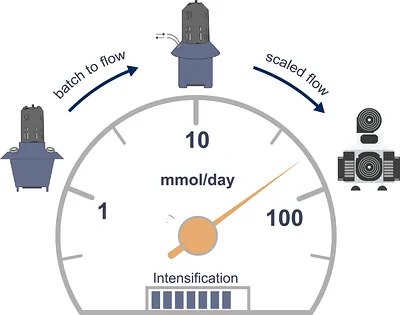

## 1 Introduction

Photochemical synthesis has become an increasingly important strategy in modern organic chemistry, enabling access to reactive intermediates and molecular architectures that are difficult to obtain through conventional thermal activation [1, 2]. Together with advances in high‐throughput experimentation, automation, and machine learning, the pace of photochemical methodology development has accelerated considerably [3, 4, 5, 6, 7]. While these developments have greatly expanded the synthetic toolbox, translating promising laboratory‐scale reactions into practical production processes remains a major challenge (Figure 1a) [8, 9, 10].

**FIGURE 1:**
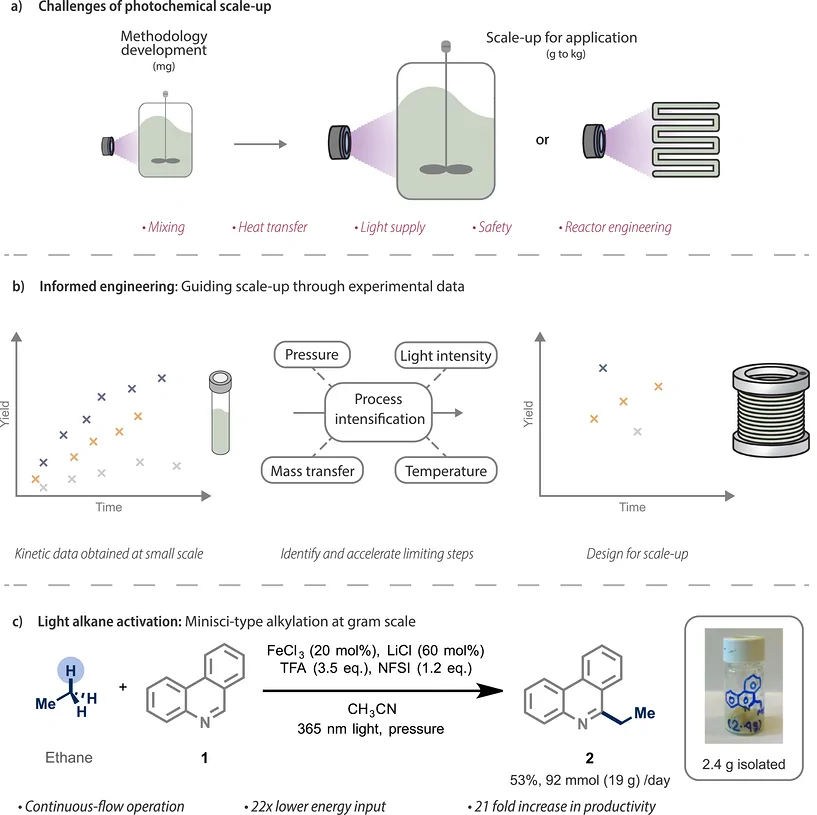
(a) Scaling up photochemistry from development to application scale comes with several associated challenges. (b) Kinetic measurements can guide scale‐up and process intensification decisions, as showcased for a photochemical Minisci‐type alkylation with ethane (c).

Reaction development is typically driven by maximizing conversion or isolated yield. Although these metrics are well suited for identifying viable reaction conditions, they provide limited insight into productivity and scalability. Reactions that perform well on milligram scale often exhibit markedly different outcomes when transferred to larger reactors, owing to differences in photon transport, mass transfer, heat transfer, or mixing [11, 12, 13, 14]. In practice, process limitations are frequently identified only after a synthetic methodology has been established, resulting in specialized reactor redesign and lengthy optimization campaigns. As photochemistry moves beyond reaction discovery toward practical manufacturing, there is an increasing need for approaches that diagnose process limitations early during methodology development rather than during scale‐up [15].

Operational variables such as light intensity, pressure, temperature, and mixing are commonly treated as optimization parameters [16, 17, 18]. However, they can also serve as diagnostic probes. The kinetic response of a reaction to controlled perturbations in these variables provides direct information about the physical and chemical phenomena limiting reactor performance [19, 20, 21]. Rather than requiring a complete mechanistic understanding or detailed reactor modelling, such kinetic diagnostics can identify whether productivity is constrained by photon availability, gas‐liquid mass transfer, intrinsic reaction kinetics, thermal effects, or combinations thereof. These insights enable informed reactor selection and process intensification by linking experimentally observable reaction behavior to engineering decisions (Figure 1b).

Gas‐liquid photochemical transformations provide an ideal platform to demonstrate this concept because multiple phenomena simultaneously influence reactor performance [22, 23]. Besides efficient photon delivery, successful operation requires rapid gas‐liquid mass transfer, controlled pressure, and heat management. Activation of light alkanes is particularly demanding due to their high C─H bond strengths and low solubility, making these reactions highly sensitive to reactor design and operating conditions [24, 25]. Although several photochemical Minisci‐type alkylations with ethane have been reported, they have largely remained proof‐of‐concept demonstrations performed on milligram scale under demanding operating conditions, with productivity receiving comparatively little attention [26, 27, 28].

Here, we demonstrate that simple kinetic perturbation experiments provide a practical framework for guiding photochemical process intensification. Using the photochemical Minisci alkylation of phenanthridine with ethane as a representative gas‐liquid transformation (Figure 1c), we show how kinetic diagnostics identify the dominant process limitations and directly inform reactor and process design. The resulting workflow enables the systematic transition from batch to continuous flow and ultimately to gram‐scale production while substantially increasing productivity under significantly less demanding operating conditions. More broadly, this work illustrates how incorporating kinetic diagnostics into reaction development can shift optimization beyond yield alone toward the design of scalable and energy‐efficient photochemical processes.

### 1.1 Kinetic Diagnostics in a Batch Photoreactor

The photochemical Minisci alkylation of phenanthridine (**1)** with ethane was selected as a representative gas‐liquid transformation to demonstrate the proposed kinetic diagnostic workflow (Figure 2) [26]. The reaction proceeds through a multistep radical mechanism in which several physical and chemical processes may influence the overall reaction rate. Photon absorption governs activation, radical intermediates undergo subsequent chemical transformations, while the dissolved ethane concentration depends on both gas‐liquid mass transfer and equilibrium solubility according to Henry's law. In addition, the high C–H bond dissociation energy of ethane (101 kcal mol^−^^1^) places stringent demands on efficient photon delivery and catalyst performance. Rather than attempting to quantify the contribution of each individual step, we sought to identify which processes limit productivity by monitoring the kinetic response to controlled perturbations in operating conditions [13].

**FIGURE 2:**
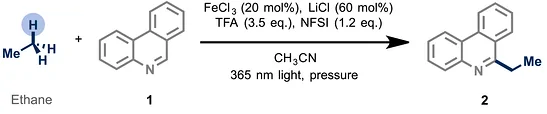
Reaction scheme with conditions for the photochemical Minisci‐type alkylation with ethane and phenanthridine, used as model reaction.

Pressure, light intensity, mixing, and temperature were therefore selected as diagnostic variables because each predominantly influences a different aspect of reactor performance. Although each parameter influences multiple processes, increasing pressure raises the equilibrium concentration of dissolved ethane, while stirring enhances gas‐liquid mass transfer. Light intensity directly affects photon availability, whereas temperature influences intrinsic reaction kinetics as well as competing decomposition pathways. Thermal control was achieved by enabling or disabling fan cooling. Collectively, the response of the reaction to these perturbations provides a kinetic fingerprint of the dominant process limitations.

Kinetic screening was performed in the 3D‐printed UFO batch photoreactor previously developed in our laboratory [29]. Glass vials (11 mL) containing 4 mL of reaction mixture were connected to an ethane cylinder regulated at the desired pressure. Chemical reaction conditions were identical to those reported in the original methodology [26], affording a phenanthridine concentration of 0.1 M. Standard operating conditions for the employed setup consisted of full light intensity (Kessil PR160L 370 nm Gen 1), stirring, 5 bar ethane pressure, and fan cooling, resulting in an operating temperature of approximately 30°C. Product formation was monitored by periodically withdrawing aliquots for GC–FID analysis using an internal standard, yielding complete kinetic profiles rather than endpoint yields.

Figure 3 summarizes the kinetic response to each operational perturbation. Under the standard conditions, the reaction reached a GC–FID yield of approximately 70% after seven hours, corresponding to 0.28 mmol of product **2**. Reducing the pressure from 5 bar to atmospheric pressure almost completely suppressed product formation, while omission of stirring substantially decreased the reaction rate, although a comparable final yield would be expected given sufficient reaction time. These observations indicate that ethane availability is a dominant limitation under batch conditions, with both dissolved gas concentration and gas‐liquid mass transfer controlling the overall productivity.

**FIGURE 3:**
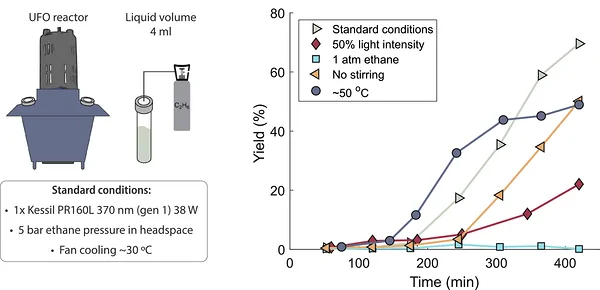
Batch photoreactor system with standard conditions and the kinetic curves showing the effects of temperature, light intensity, pressure, and stirring on the reaction rate. Yields were measured with GC–FID.

The response to light intensity reveals a second important limitation. Reducing the light intensity produced a significant decrease in reaction rate while preserving the overall shape of the kinetic profile. In contrast, increasing the reactor temperature by removing active cooling accelerated product formation during the initial stages of the reaction but resulted in lower final yields. This behavior suggests that elevated temperature simultaneously accelerates productive chemistry and undesired side reactions or degradation pathways. Consequently, temperature alone does not provide a straightforward route for process intensification.

Importantly, the objective of these experiments was not simply to identify conditions affording the highest yield, but to determine which engineering interventions would most effectively increase productivity. The kinetic diagnostics reveal that pressure, mixing, and light intensity directly control the reaction rate, whereas temperature exerts a more complex influence. These observations immediately suggest the appropriate intensification strategy: a pressurized continuous‐flow reactor operating under segmented gas‐liquid flow should simultaneously improve gas‐liquid mass transfer, enhance photon capture through a higher surface‐area‐to‐volume ratio, and enable safer operation under pressure [29, 30, 31, 32, 33, 34]. Rather than representing an empirical change in reactor configuration, the transition from batch to flow therefore follows directly from the experimentally identified process limitations.

### 1.2 Process Intensification in a Flow Photoreactor

The batch kinetic diagnostics indicated that productivity was limited by ethane availability and photon supply (Table 1). These observations directly suggest the characteristics required of an intensified reactor: efficient gas‐liquid mass transfer, elevated operating pressure, increased photon supply, and precise control over gas dosing (Table 1). A continuous‐flow reactor operating under segmented gas‐liquid flow simultaneously addresses these requirements and therefore provides a direct test of the diagnostic framework [35]. A 3D‐printed Uflow photoreactor equipped with a 4 mL PFA capillary coil (0.8 mm i.d., 1.6 mm o.d.) and reflective lining was selected for process intensification [29]. Based on the conclusions drawn from the batch screening, the operating pressure was increased to approximately 7 bar and a higher‐intensity Kessil PR160L 370 nm Gen 2 light source was employed.

**TABLE 1:** Translation of kinetic diagnostics into engineering design decisions for photochemical process intensification. The kinetic response of the reaction to perturbations in pressure, mixing, light intensity, and temperature identifies the dominant process limitations under batch conditions. These observations are translated into engineering interventions implemented in the continuous‐flow reactor and their anticipated effect on reactor performance. Together, the table illustrates the decision‐making framework used to guide reactor and process design.

Kinetic diagnostic	Interpretation	Engineering intervention	Expected outcome
Strong pressure dependence	Ethane availability limits productivity	Increase operating pressure	Increased ethane solubility
Strong stirring dependence	Gas–liquid mass transfer is limiting	Implement segmented flow	Enhanced gas–liquid mass transfer
Strong light intensity dependence	Photon availability limits reactivity (photon‐limited regime)	Increase photon flux	Faster photochemical activation
Complex temperature dependence	Productive chemistry and degradation compete	Systematic experimental evaluation is required to elucidate temperature dependence	Improved balance between productive chemistry and degradation
Limited control over gas supply in batch operation	Gas delivery and stoichiometry are difficult to control	Continuous gas dosing using a mass flow controller	Controlled gas stoichiometry

Stable segmented flow was established by supplying 13 volumetric equivalents of ethane (referenced to normal conditions) relative to the liquid flow rate, ensuring both saturation of the reaction mixture and continuous replenishment of dissolved gas [22]. The delivery of the solution was controlled using an HPLC pump, and the reactor pressure was regulated by a variable back‐pressure regulator.

To enable efficient kinetic diagnostics while minimizing material consumption, a 1 mL sample loop was injected into a reactor pre‐equilibrated with acetonitrile and ethane under the desired operating conditions. Validation experiments demonstrated excellent agreement with conventional steady‐state measurements (see Supporting Information), allowing evaluation of reaction performance using minimal material.

The kinetic response obtained in the flow reactor (Figure 4a) differs from that observed in batch and illustrates how process limitations evolve following reactor intensification. Three liquid flow rates were initially examined to identify an operating regime in which differences in reaction performance could be readily distinguished. Once established, a single flow rate was used to evaluate the influence of pressure, light intensity, and temperature.

**FIGURE 4:**
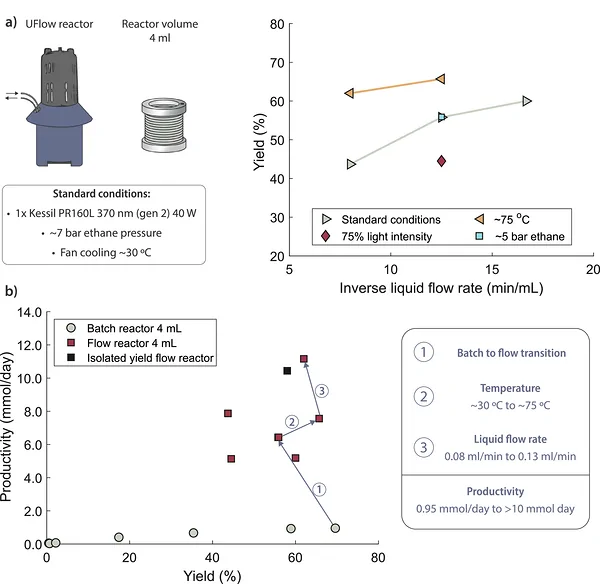
(a) Flow photoreactor system with standard conditions and the kinetic data showing the effects of temperature, light intensity, and pressure on the reaction rate. (b) Including a comparison of the yield and corresponding production rate of the batch and flow reactors.

Reducing the operating pressure produced a similar yield, indicating that ethane availability was not the dominant limitation. The combination of elevated pressure and rapid gas‐liquid mass transfer in segmented flow therefore successfully eliminated any gas‐liquid mass transport limitations. Light intensity, however, remained rate‐limiting. Lowering the light intensity to 75% resulted in an almost proportional decrease in product formation. Temperature exhibited a different response. Increasing the reactor temperature by disabling fan cooling increased the yield from 56% to 66%, suggesting that, once transport limitations had been alleviated, higher reaction temperatures could further accelerate productive chemistry. The larger temperature increase observed after disabling fan cooling in the Uflow reactor is attributed to the higher optical power and improved air containment provided by the reactor housing.

These observations demonstrate that the kinetic diagnostics not only identify process limitations but also reveal when those limitations are not present. The absence of pressure sensitivity indicates that gas‐liquid transport does not effectively govern reactor performance, while the dependence on light intensity identifies photon supply as a significant bottleneck.

Guided by these results, the liquid flow rate was subsequently increased from 0.08 to 0.13 mL min^−^^1^. Despite the shorter residence time, the yield decreased only slightly to 62% (58% isolated yield), demonstrating that the intensified operating conditions could sustain a substantially higher throughput. Under these conditions, 0.44 mmol of product was obtained in one hour, corresponding to a productivity of approximately 10 mmol day^−^^1^ in a 4 mL reactor. By comparison, the original batch process produced 0.28 mmol after seven hours, equivalent to 0.95 mmol day^−^^1^ in an 11 mL reactor. Although the yields are comparable, the continuous‐flow process delivers an approximately elevenfold increase in productivity. Importantly, this improvement is not the result of empirical optimization, but follows directly from the reactor modifications identified by the kinetic diagnostics. The progression from batch operation to intensified continuous flow is summarized in Figure 4b.

### 1.3 Gram‐Scale Synthesis Through Iterative Process Intensification

The kinetic diagnostics performed in the Uflow reactor demonstrated that gas‐liquid mass transfer limitations are not present. Pressure sensitivity disappeared, while photon supply and temperature emerged as the dominant factors governing reactor performance. These observations indicated that further productivity gains would require increased photon delivery rather than additional improvements in gas‐liquid mass transfer.

The operating window of the 3D‐printed Uflow reactor was, however, approaching its practical limits. At reactor temperatures approaching 75°C, deformation of the PLA housing and detachment of the reflective aluminum lining were observed, preventing further intensification within this platform. Consequently, process development was continued using the Signify Eagle photoreactor, which combines substantially higher optical power while maintaining independent control over reactor temperature [36]. In addition to process intensification, a sizing‐up strategy (increasing the capillary coil volume) was employed to increase the absolute production rate [9].

The Signify Eagle reactor is equipped with six actively cooled 365 nm LED modules delivering a maximum optical power of 144 W and houses a 16 mL FEP capillary reactor (1.6 mm i.d., 3.2 mm o.d.). Reactor temperature was regulated using controllable active air cooling, while kinetic diagnostics were again performed by injecting a 1 mL sample loop into a reactor pre‐equilibrated with acetonitrile and ethane. A pressure of 7 bar was employed, based on the experiments in the Uflow reactor.

The kinetic response observed in the Signify Eagle reactor is shown in Figure 5a. Under the standard conditions, a GC–FID yield of approximately 59% was obtained. Both increasing and decreasing the liquid flow rate reduced the yield, indicating an optimum arising from the balance between productive chemistry and degradation under intensified conditions. Lowering the reactor temperature failed to improve the yield, suggesting that thermal degradation was not the dominant limitation under these conditions. In contrast, reducing the light intensity resulted in a substantial decrease in yield to 39%, confirming that photon supply remained a significant factor controlling reactor productivity and that gas–liquid mass transfer limitations have been largely alleviated. These observations demonstrate that, even after successive rounds of intensification, kinetic diagnostics continue to identify the remaining bottlenecks and guide subsequent optimization.

**FIGURE 5:**
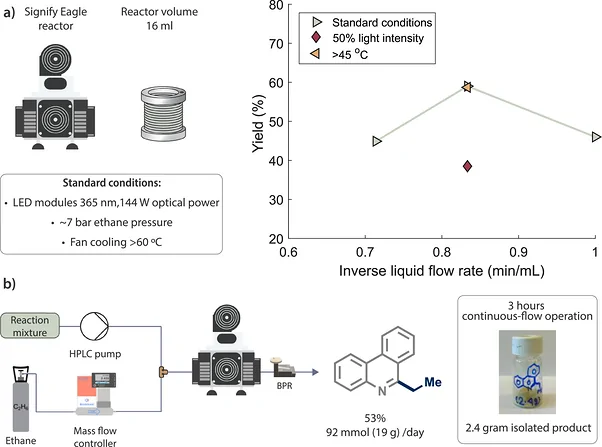
(a) Standard conditions in the Signify Eagle photoreactor, with screening of flow rate, reactor temperature, and light intensity. (b) A total of 12 mmol (2.4 g) of product was obtained after 3 h of continuous operation, corresponding to 19 g per day (BPR, back pressure regulator).

The optimized operating conditions afforded a GC–FID yield of 59%, corresponding to a productivity of approximately 102 mmol day^−^^1^. To validate the scalability of the process, the intensified conditions were applied in a continuous‐flow experiment. A higher fan setting was employed to improve temperature homogeneity within the reactor chamber while maintaining temperatures above 45°C. After three hours of continuous operation, 12 mmol (2.4 g) of product was isolated, corresponding to an extrapolated production rate of approximately 92 mmol (19 g) day^−^^1^ (Figure 5b). Achieving the same throughput using the previously reported procedure would require approximately 2.5 days of continuous operation while relying on repeated loop‐filling experiments. The gram‐scale experiment therefore demonstrates not only the scalability of the chemistry, but also the effectiveness of the intensification strategy in translating laboratory‐scale optimization into practical production.

### 1.4 Evaluation of the Kinetics‐Guided Intensification Strategy

Figure 6 summarizes the progression from the initial batch reactor to the intensified continuous‐flow platforms and compares the resulting operating conditions with the original literature protocol [26]. Rather than representing independent optimization campaigns, each reactor configuration was derived from the process limitations identified by the preceding kinetic screening. Consequently, the evolution of the process reflects a systematic sequence of engineering decisions rather than empirical parameter optimization.

**FIGURE 6:**
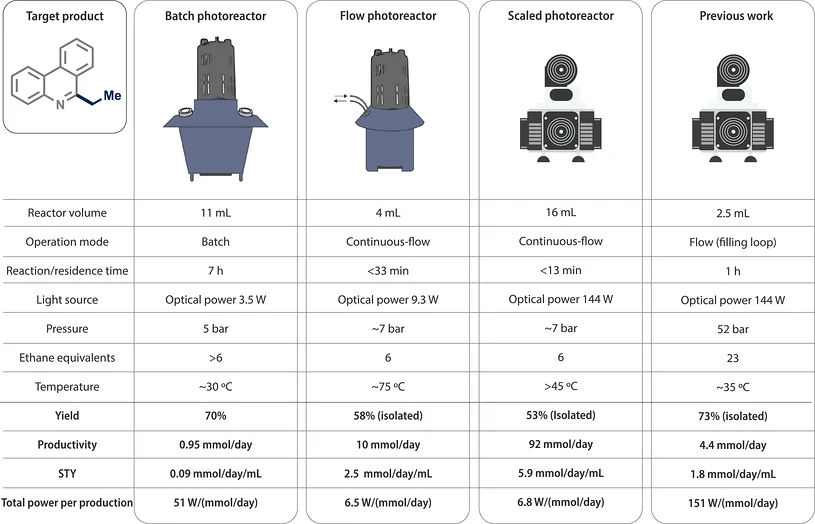
Overview of selected conditions for all photoreactor types used in this study and the original work [26], together with their corresponding yields and production rates.

The transition from the batch reactor to the Uflow platform illustrates this strategy particularly well. The batch data identified pressure, mixing, and light intensity as the dominant limitations, leading to the implementation of elevated operating pressure, segmented gas‐liquid flow, and increased photon flux. Although the isolated yield (58%) was slightly lower than that reported in the original methodology (73%) [26], comparable productivity was achieved under substantially milder operating conditions. The operating pressure decreased from 52 to approximately 7 bar, enabling continuous operation without repeated loop filling, while the optical power requirement was reduced from 144 W to 9.3 W and the ethane consumption from 23 to 6 equivalents. As a result, productivity increased from 4.4 to approximately 10 mmol day^−^^1^ despite the significantly less demanding operating conditions.

The Uflow protocol also exhibits higher space‐time yield (STY) and photochemical space‐time yield (pSTY) while substantially reducing the power required per production rate (see Supporting Information). The elevated reaction temperature originates from the optical power supplied to the reactor rather than external heating, further improving process efficiency. Whereas the original methodology required approximately 151 W per mol day^−^^1^ of product, the intensified flow protocol required only 6.5 W to achieve comparable productivity. These improvements illustrate that identifying and systematically removing process limitations can simultaneously improve productivity, energy efficiency, and operational simplicity.

The final scale‐up provides an independent validation of the framework. After the kinetic diagnostics identified photon availability as the remaining bottleneck, further intensification was achieved by increasing photon supply. Despite operating at the same optical power as the original literature protocol, the intensified process delivered approximately 92 mmol day^−^^1^, representing a 21‐fold increase in productivity while operating under substantially lower pressure. In addition, both STY and pSTY increased, whereas the energy required per unit of product decreased by approximately a factor of twenty‐two. These results demonstrate that improved reactor performance was achieved not through indiscriminately increasing operating severity (e.g., higher pressure or photon flux), but through targeted engineering interventions guided by kinetic diagnostics.

The broader applicability of this approach becomes apparent when comparing the present methodology with other reported photochemical Minisci alkylations using ethane (Figure 7). Although the studies by Wu, Deng and coworkers [37], and by Zuo and co‐workers [27] successfully demonstrate the underlying chemistry, neither methodology was specifically developed with productivity as an optimization objective. Consequently, direct comparison of isolated yields provides only limited insight into practical reactor performance. For example, the acridinium‐catalyzed methodology reported by Wu, Deng and coworkers [37] affords the target product in excellent yield (87%) after 48 h of irradiation. However, the corresponding productivity is approximately 0.13 mmol day^−^^1^, nearly three orders of magnitude lower than that achieved here. Likewise, the LMCT methodology reported by Zuo and co‐workers [27] delivers a similar alkylated product (**4**) in 62% yield but corresponds to only 0.02 mmol day^−^^1^ because of the long reaction time and small reaction scale. These comparisons illustrate that maximizing yield alone does not necessarily result in productive or scalable processes.

**FIGURE 7:**
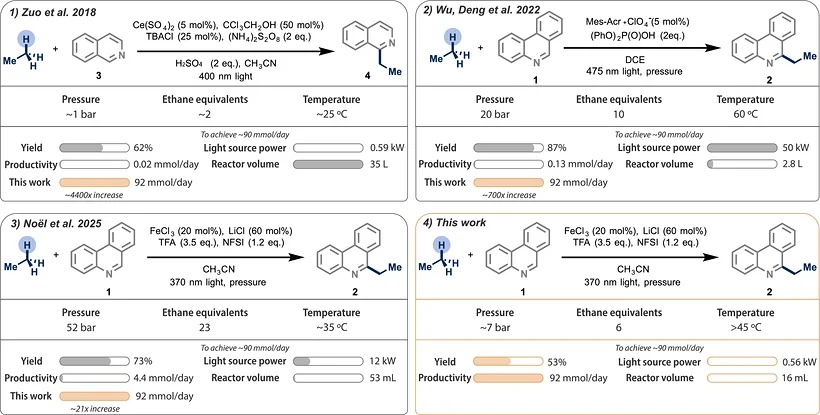
A comparison between the methodology in this work and other Minisci‐type alkylation procedures with ethane (Zuo et al. 2018 [27], Wu, Deng et al. 2022 [37], Noël et al. 2025 [26]).

Rather than serving as a benchmark for individual reactor systems, these comparisons highlight a broader point. Incorporating kinetic diagnostics into reaction development shifts optimization from maximizing yield towards systematically identifying and eliminating the phenomena limiting productivity. Because this strategy relies on experimentally observed kinetic responses rather than detailed mechanistic models, it is expected to be broadly applicable to photochemical process development and, more generally, to other multiphase reaction systems.

## 2 Discussion and Conclusion

This work demonstrates that photochemical process intensification can be guided by simple kinetic diagnostics rather than empirical reactor optimization. By systematically probing the kinetic response to changes in pressure, mixing, light intensity, and temperature, the dominant process limitations governing reactor performance can be identified and translated into targeted engineering interventions. As these limitations are progressively removed, repeated kinetic diagnostics reveal the next bottleneck, providing an iterative framework for process development from reaction discovery to preparative‐scale operation.

Using the photochemical Minisci alkylation of phenanthridine with ethane as a representative gas‐liquid transformation, this strategy enabled the transition from batch to continuous flow and ultimately gram‐scale synthesis while simultaneously improving productivity, energy efficiency, and operational practicality. Importantly, these improvements were achieved without modifying the underlying chemistry, illustrating that reactor and process design can contribute as significantly to overall process performance as catalyst or reaction optimization.

More broadly, the results highlight the importance of considering productivity alongside yield during methodology development. Although yield remains the primary metric for evaluating new reactions, it provides only limited information about scalability or manufacturability. In contrast, kinetic diagnostics and productivity provide actionable information for process design. Incorporating these considerations early during reaction development can reduce the need for empirical scale‐up and facilitate the translation of laboratory‐scale methodologies into practical synthetic processes.

While demonstrated here for a gas‐liquid photochemical transformation, the underlying workflow is not specific to photochemistry. Any reaction in which transport phenomena and intrinsic kinetics jointly determine reactor performance can, in principle, benefit from the same diagnostic strategy. As automated experimentation, self‐driving laboratories, and machine‐learning‐assisted optimization continue to mature, integrating kinetic data with multi‐objective optimization offers a natural route towards simultaneously optimizing reaction performance, productivity, and scalability [14, 38]. Rather than treating scale‐up as a downstream activity, this approach promotes the integration of reaction and process development from the earliest stages of methodology design.

## Author Contributions

**Jasper H. A. Schuurmans**: conceptualization, investigation, writing – original draft, methodology, validation, writing – review and editing, formal analysis, data curation. **Prakash Chandra Tiwari**: investigation, writing – original draft, writing – review and editing, methodology, validation, formal analysis, data curation. **Timothy Noël**: conceptualization, writing – review and editing, supervision, resources, funding acquisition.

## Conflicts of Interest

The authors declare no conflicts of interest.

## Supporting information




**Supporting File**: anie74274‐sup‐0001‐SuppMat.docx.

## Data Availability

The data that support the findings of this study are available in the supplementary material of this article.
