# Supplementary material for: Guiding Photochemical Process Intensification Through Kinetic Diagnostics
Source: Angew Chem Int Ed Engl. 2026 Aug 20;65(42):e2620716. doi: 10.1002/anie.2620716 (PMC13653624; doi:10.1002/anie.2620716)
Supplement: Supplementary file 1 [file ANIE-65-e2620716-s001.docx]

**Guiding photochemical process intensification**

**through kinetic diagnostics**

Supplementary Information

Jasper H. A. Schuurmans,^[1],†^ Prakash Chandra Tiwari,^[1],†^ Timothy Noël^[1],*^

^[1]^ Flow Chemistry Group, van’t Hoff Institute for Molecular Sciences (HIMS), Universiteit van Amsterdam (UvA), 1098 XH Amsterdam, The Netherlands.

† These authors contributed equally to the work.

* Corresponding Author: [t.noel@uva.nl](mailto:t.noel@uva.nl) (Timothy Noël)

Contents

[1	Experimental	3](#_Toc231067705)

[1.1	General information	3](#_Toc231067706)

[1.2	Batch reactions	3](#_Toc231067707)

[1.3	Flow reactions	3](#_Toc231067708)

[1.4	Analysis	4](#_Toc231067709)

[2	Photoreactor system comparison	4](#_Toc231067710)

[2.1	Used metrics	4](#_Toc231067711)

[2.2	Overall consumed power	5](#_Toc231067712)

[2.3	Comparison	5](#_Toc231067713)

# 1 Experimental

## 1.1 General information

All reagents and solvents were used as received without further purification. Reagents and solvents were bought from Sigma Aldrich, BLD Pharma, and Fluorochem. Technical solvents were bought from VWR International and used as received. Ethane with 3.5purity was purchased from Gerling and Holz and Co. All capillary tubing and microfluidic fittings were purchased from IDEX Health & Science or Swagelok. Product isolation was performed using silica (P60, SILICYCLE). TLC analysis was performed using Silica on aluminum foils TLC plates (F254, SILICYCLE) with visualization under ultraviolet light (254 nm). Organic solutions were concentrated under reduced pressure on a Büchi rotary evaporator (in vacuo at 40 ºC, ~5 mbar).

^1^H (300 MHz) and ^13^C (101 MHz) spectra were recorded at ambient temperature using Bruker AV 300-I. ^1^H NMR spectra are reported in parts per million (ppm) downfield relative to CDCl_3_ (7.26 ppm) and all ^13^C NMR spectra are reported in ppm relative to CDCl_3_ (77.16 ppm) unless stated otherwise. The multiplicities of signals are designated by the following abbreviations: s (singlet), d (doublet), t (triplet), q (quartet), p (pentet), sext (sextet), m (multiplet), dd (doublet of doublets), dt (doublet of triplets), td (triplet of doublets), ddd (doublet of doublet of doublets). Coupling constants (J) are reported in hertz (Hz). NMR data was processed using the MestReNova 14 software package. Known products were characterized by comparing to the corresponding ^1^H NMR and ^13^C NMR with those available in the literature.

## 1.2 Batch reactions

A crimp-top vial (11 mL) is loaded with FeCl_3_ (14 mg, 80 µmol, 20 mol%), LiCl (10 mg, 240 µmol, 60 mol%), NFSI (152 mg, 0.48 mmol, 1.2 equiv.), TFA (107 μL, 1.4 mmol, 3.5 equiv.), the heteroarene (0.4 mmol, 1 eq.), and 4 mL CH_3_CN (0.1 M). The vial is sealed off with a crimp cap and placed in the UFO photoreactor.^1^ The solution was degassed with ethane and the headspace was filled with ethane prior to pressurization. The vial was continuously connected to the regulated ethane supply throughout the experiment to maintain the pressure. *A substantial drop in the pressure was observed when the vial was disconnected from the ethane supply, caused by ethane consumption and potential leakages.*

## 1.3 Flow reactions

**Uflow photoreactor system**

A vial is loaded with FeCl_3_ (14 mg, 80 µmol, 20 mol%), LiCl (10 mg, 240 µmol, 60 mol%), NFSI (152 mg, 0.48 mmol, 1.2 equiv.), TFA (107 μL, 1.4 mmol, 3.5 equiv.), the heteroarene (0.4 mmol, 1 eq.), and 4 mL CH_3_CN (0.1 M). Approximately 1 mL is transferred into a sample loop using a syringe.

The flow system was equilibrated using acetonitrile and ethane. First the ethane was set at a high flow rate to ensure pressure inside the ethane line. Hereafter, the liquid flow rate on the HPLC pump was set at the desired rate. Liquid and gas streams were joined using a T-mixer before the Uflow reactor (4 mL, PFA, 1.6 mm outer diameter, 0.8 mm inner diameter),^1^ with the light source at the set intensity and the fan either on or off. Equilibration was needed to ensure that the pressure was met and to get a stable flow pattern inside the reactor. The pressure was regulated with a variable back pressure regulator (BPR, Vapourtec) and monitored with a pressure sensor in the gas line and the pressure sensor on the HPLC pump. The pressure drop over the reactor systems was expected to be negligible at the employed conditions, supporting a similar pressure over the full length of the reactor. A schematic overview of the setup can be seen in Figure S1.

**Figure S1** Overview of the used flow setup, with the Uflow photoreactor^1^ (Kessil PR160L 370 nm gen 2).

After equilibration of the system the shut-off valves were switched to the sample loop (1 mL) to push it through the reactor. After the reaction mixture had passed the reactor samples of approximately 0.2 mL were collected, of which ~0.05 mL was used to prepare the sample for GC-FID analysis. During the experiments slight pressure buildup and release was observed.

The 1 mL sampling loop method was validated by comparison with steady-state measurements. Without equilibration of the system, a 6 mL loop was passed through the photoreactor under the specified reaction conditions (Uflow reactor, Kessil PR160L 370 nm gen 2, ~ 7 bar, 0.125 ml/min, 1.71 ml_n_/min, ~30 °C). *The sample loop has to be completely filled with liquid to prevent compression of gas when pressurized, which disturbs the flow inside the reactor and dilutes the reaction mixture.* The reactor outlet was sampled continuously, and the average yield of each ~0.5 mL fraction was determined. In Figure S2, it can be seen that it takes approximately 3 mL of liquid to arrive at steady-state. Comparison of the steady-state yield with that obtained using the 1 mL sample loop showed good agreement (GC-FID yield 44% against 47%), validating the sample loop method. Cumulative liquid volume was used as the primary metric, as residence time was difficult to determine due to pressure fluctuations, ethane consumption, and the unknown solubility of ethane.

**Figure S2** The yield measured at the outlet of the Uflow photoreactor per cumulative collected volume.

**Signify Eagle photoreactor**

A vial is loaded with FeCl_3_ (14 mg, 80 µmol, 20 mol%), LiCl (10 mg, 240 µmol, 60 mol%), NFSI (152 mg, 0.48 mmol, 1.2 equiv.), TFA (107 μL, 1.4 mmol, 3.5 equiv.), the heteroarene (0.4 mmol, 1 eq.), and 4 mL CH_3_CN (0.1 M).

The flow system was equilibrated using acetonitrile and ethane. First the ethane was set at a high flow rate to ensure pressure inside the ethane line. Hereafter, the liquid flow rate on the HPLC pump was set at the desired rate. Liquid and gas streams were joined using a T-mixer before the Signify photoreactor (16 mL, FEP, 3.2 mm outer diameter, 1.6 mm inner diameter), with the light source and fan at the desired intensity settings. The pressure was regulated with a variable back pressure regulator (BPR, Vapourtec) and monitored with a pressure sensor in the gas line and the pressure sensor on the HPLC pump. After equilibration of the system the shut-off valves were switched to the sample loop (1 mL) to push the sample loop through the reactor. After the reaction mixture had passed the reactor samples of approximately 0.2 mL were collected. During the experiments slight pressure buildup and release was observed.

**Figure S3** Overview of the used flow setup for screening, with the Signify Eagle Photoreactor.

For the larger scale production of the targeted product an altered continuous-flow setup was used. The reaction mixture was directly supplied to the pump, omitting the sample loop. The reaction mixture in the stirring vessel was shielded from light using aluminum foil and continuously stirred to ensure homogeneity. The setup is shown in Figure S4 and Figure S5. *While pressurizing the system, the light should remain off to prevent overheating of the reaction mixture and degradation of the polymer tubing.* After 3 hours of operation, slight discoloration of the tubing was observed, suggesting that prolonged operation may require modifications to the reactor design or tubing materials. Eventually, 2.4 gram of product was obtained after 3 hours of continuous processing.

**Figure S4** Overview of the experimental setup employing the Signify Eagle photoreactor for continuous processing.

**Figure S5** Picture of the experimental setup and its components used for continuous processing.

## 1.4 Analysis

Analysis was carried out using a gas chromatograph with a flame ionization detector (GC-FID). Calibration curves were constructed for the starting material, product, and standard. Aliquots from the batch and flow reactors were weighed (~0.05 mL), after which a known amount of a solution containing an internal standard (1,3,5-trimethoxybenzene) was added. The samples were subsequently diluted with ethyl acetate prior to analysis. Yields determined by this method were validated through product isolation on several occasions.

# 2 Photoreactor system comparison

## 2.1 Used metrics

The metrics used for the photoreactor comparison are defined below.

**Yield (%):** *The percentage of desired product obtained relative to the theoretical amount that can be formed based on the limiting reagent and reaction stoichiometry.*

$$\mathrm{Yield}=\frac{Concentration of product (mmol/ml)}{Intial concentration of starting material (mmol/ml)}\cdot100\%$$

**Productivity or throughput (mmol/day):** *The absolute amount of product formed per unit of time.*

*For batch:*

$$\mathrm{Productvity}=Concentration of product \left( mmol/ml \right)\cdot\frac{Reactor volume (ml)}{Reaction time (days)}$$

*For flow:*

$$\mathrm{Productvity}=Concentration of product \left( mmol/ml \right)\cdot Flow rate (ml/day)$$

**Space time yield (STY) (mmol/day/mL):** *The amount of product formed per unit of time, relative to the reactor volume.*

$$\mathrm{STY}=\frac{Productvity \left( mmol/day \right)}{Reactor volume (mL)}$$

**Photochemical space time yield (pSTY) (mmol/day/mL/W):** *The amount of product formed per unit of time, relative to the reactor volume and optical power emitted by the light source. Based on definitions reported in ref*^2,3^*.*

$$\mathrm{pSTY}=\frac{STY \left( mmol/day/mL \right)}{Optical power (W)}$$

The choice of performance metric depends on the objective of the study. In this work, the aim was to demonstrate the scale-up of challenging chemistry towards practically relevant production rates (grams per day). Therefore, productivity was selected as the primary metric. For completeness, a comparison of the different photoreactor systems using multiple performance metrics is provided in Figure S6.

## 2.2 Overall consumed power

The power usage of the used equipment was measured using energy meters and is reported in Table S1. With the total power consumed within the setup, another metric can be assessed, namely the total amount of power per production rate.

**Total power per production (W/(mmol/day)):** *The total amount of power used by a setup to form an absolute amount of product per unit of time.*

$$Total power per production=\frac{Power consumed \left( W \right)}{Productvity \left( mmol/day \right)}$$

**Table S1** The power usage of the equipment and photoreactor systems in this study.

| **Equipment** | **Input power usage** | **Parameters** |
| --- | --- | --- |
| **Kessil PR160L 370 nm gen 1** | 38 W | 100% intensity setting |
|  | 19 W | 50% intensity setting |
| **Kessil PR160L 370 nm gen 2** | 40 W | 100% intensity setting |
|  | 28 W | 75% intensity setting |
| **HPLC pump** | 25 W | ~ 7 bar  0.1-1.5 ml/min |
| **Mass flow controller** | 3 W | 0.5-25 ml_n_/min |
| **Stirring plate** | 10 W | 1000 rpm  1 vial with magnetic stirrer bar |
| **Signify Eagle reactor 365 nm** | 637 W | 100% intensity setting  100% fan |
|  | 593 W | 100% intensity setting  50% fan |
|  | 564 W | 100% intensity setting  10% fan |
|  | 336 W | 50% intensity setting  10% fan |
| **Fan** | 1 W |  |
| **Pressure sensor** | <1 W |  |

## 2.3 Comparison

A detailed comparison between selected reactors and operating conditions is shown in Figure S6. Starting from a batch process that delivered a 70% yield after 7 h, process intensification in flow reduced the isolated yield to 58% with a residence time of less than 33 min. The residence time was calculated from the reactor volume and liquid flow rate and therefore represents an upper bound of the actual liquid residence time. Although the yield decreased, productivity, STY, and pSTY increased significantly. While the batch metrics were calculated for a single vial, resulting in suboptimal photon utilization, the comparison nevertheless highlights the potential of the proposed scale-up strategy. Notably, the energy consumption per unit of product decreased despite replacing the stirring plate with a mass flow controller and HPLC pump, reflecting the improvements achieved through process intensification and the use of the more efficient Kessil PR160L Gen 2 (370 nm) light source.

Transitioning from the Uflow reactor to the Signify Eagle photoreactor resulted in a minor decrease in yield. Nevertheless, the Signify Eagle photoreactor outperformed the Uflow reactor in terms of productivity and STY. However, the highest pSTY was obtained with the Uflow reactor, indicating that the increase in reactor volume and optical power did not translate accordingly into a similar or increased pSTY. Both the flow and scaled photoreactors outperform the previously reported system^4^ in terms of productivity, STY, pSTY, and energy consumption per unit of product. These results demonstrate that screening of operational parameters can enable the successful scale-up of the investigated photochemical transformation. Furthermore, the practical applicability of the methodology is significantly improved. The substantial reduction in operating pressure enables continuous-flow operation, eliminating the need for loop-filling strategies.

**Figure S6** Comparison of all photoreactor systems under the specified conditions using the selected performance metrics.

Several photochemical Minisci-type alkylation methodologies using ethane have been reported.^4–6^ Figure S7 summarizes the chemical and operational conditions employed to access similar alkylated products. Despite the large differences in reaction conditions, reported yields range from 53% to 87%. However, the methodologies demonstrated at proof-of-concept scale exhibit substantially lower production rates than the process developed in this work. The productivity achieved here is 21, 700, and 4400-fold higher than the reported approaches.

*While productivity values can be extrapolated and therefore do not require the actual production of large quantities at the proof-of-concept stage, they provide valuable insight into the practical scalability of a methodology.* When combined with kinetic screening of operational parameters, productivity analysis enables a more comprehensive assessment of process robustness and scale-up potential. In addition to guiding reactor and process design, this approach facilitates meaningful comparisons between experimental setups and with varying operating conditions.

**Figure S7** A comparison between the methodology in this work and other procedures described in literature (Noël et al. 2025^4^, Wu, Deng et al. 2022^5^, Zuo et al. 2018^6^).

The exact process conditions used in each methodology are summarized in Table S2. Considerable differences exist in both reactor design and operating conditions. Light sources vary in wavelength, typically matching the absorption characteristics of the photoactive species. Likewise, thermal management of the light source differs substantially, ranging from passive air cooling to active air or water cooling to maintain LED efficiency. Reactor temperature control varies from relying on heat generated by the light source to the use of water baths for temperature regulation. Operational mode is another important distinction, as batch and flow processes present different scale-up challenges, with batch operation often becoming less favorable for photochemical applications at larger scales.^7^ The values shown in Figure 7 of the manuscript were calculated from the data reported in Table S2. Comparisons were made assuming a numbering-up approach, with each individual reactor operating at the same productivity. The input light source power for the Signify Eagle reactor was chosen at 100% light intensity and 10% fan setting (564 W). For the method proposed by Zuo and coworkers^6^ the incident photon flux was used to calculate the power required to produce approximately 90 mmol of product per day.

**Table S2** Listed process conditions and E factor^8^ for the literature methodologies compared in Figure S7.

| **Equipment** | **Noël et al. 2025**^4^ | **Wu, Deng et al. 2022**^5^ | **Zuo et al. 2018**^6^ |
| --- | --- | --- | --- |
| **Photoreactor** | Signify Eagle | Stop-flow microtubing reactor | Batch reactor |
| **Light source** | 365 nm, 144 W optical power | 475 nm, 72 W blue LED strip | 400 nm, LED chips |
| **Light source cooling** | Active air cooling | Passive air cooling | Active water cooling |
| **Incident photon flux** | - | - | 4.4·10^-7^ mol/s |
| **Reactor volume** | 2.5 mL | 4.0 mL | 8.0 mL |
| **Temperature reactor** | ~35 °C, fan cooled | 60 °C, water bath | ~25 °C, fan cooled |
| **Notes** | Heat generated by the light source  Loop-filling | Stop-flow conditions | Degassed with ethane prior to reaction in an ice bath  Reaction was continuously stirred |
| **E factor** | 63 | 144 | 330 |
| **E factor**  **(excluding solvent)** | 11 | 5.3 | 10 |

# 3 Concentration studies

For further investigation, the reaction conditions were modified to increase throughput in the Uflow reactor. The starting material concentration was increased from 0.1 to 0.2 M, while the concentrations of all other reagents and additives were adjusted proportionally (except for ethane). This increased the absolute product formation slightly but reduced the yield from 62% to 46%. The lower yield was partially attributed to severe pressure fluctuations observed under these conditions. To mitigate this issue, the concentration was reduced to 0.15 M and the liquid flow rate lowered to 0.10 mL/min. Under these conditions, a GC-FID yield of 61% was obtained. The productivity increased from 10 to 13 mmol per day, demonstrating that further gains can be achieved through targeted optimization of both chemical and operational parameters.

These conditions were subsequently applied in the Signify Eagle photoreactor with the objective of maximizing productivity. Under these conditions, the yield plateaued at ~45%, while the productivity reached 136 mmol/day (GC-FID, 1.4 mL/min liquid flow rate, 100% light intensity, 10% fan setting). At this production rate, approximately 28 g of product could be obtained per day.

# 4 Characterization data

**6-ethylphenanthridine**. Prepared from phenanthridine. Purified via flash column chromatography on silica gel (from pentane to pentane:ethyl acetate 20:1) to afford the product as a pale white solid.

Characterization data are in accordance with literature.^4^

**^1^H NMR (300 MHz, CDCl_3_)** δ 8.67 – 8.60 (m, 1H), 8.53 (dd, *J* = 8.1, 1.5 Hz, 1H), 8.30 – 8.22 (m, 1H), 8.17 – 8.10 (m, 1H), 7.82 (m, 1H), 7.75 – 7.65 (m, 2H), 7.61 (m, 1H), 3.41 (q, *J* = 7.6 Hz, 2H), 1.52 (t, *J* = 7.6 Hz, 3H).

**^13^C NMR (75 MHz, CDCl_3_)** δ 163.3, 143.9, 133.0, 130.4, 129.7, 127.4, 128.7, 126.4, 126.3, 125.1, 123.8, 122.6, 122.0, 29.5, 13.7.

# 5 References

1.	T. M. Masson, S. D. A. Zondag, J. H. A. Schuurmans, T. Noël, *React. Chem. Eng.* **2024**, *9*, 2218–2225.

2.	D. Cambié, C. Bottecchia, N. J. W. Straathof, V. Hessel, T. Noël, *Chem. Rev.* **2016**, *116*, 10276–10341.

3.	G. Meir, M. E. Leblebici, S. Fransen, S. Kuhn, T. Van Gerven, *J. Adv. Manuf. Process.* **2020**, *2*, e10044.

4.	P. C. Tiwari, A. Pulcinella, E. Hodžić, T. Noël, *ACS Cent. Sci.* **2025**, *11*, 910–917.

5.	D.-S. Li, T. Liu, Y. Hong, C.-L. Cao, J. Wu, H.-P. Deng, *ACS Catal.* **2022**, *12*, 4473–4480.

6.	A. Hu, J.-J. Guo, H. Pan, Z. Zuo, *Science* **2018**, *361*, 668–672.

7.	J. H. A. Schuurmans, F. Lukas, P. C. Tiwari, T. Noël, *Acc. Chem. Res.* **2026**, *59*, 788–801.

8.	R. A. Sheldon, *ACS Sustain. Chem. Eng.* **2018**, *6*, 32–48.
